# Genome-Wide Analysis Reveals the Secondary Metabolome in *Streptomyces kanasensis* ZX01

**DOI:** 10.3390/genes8120346

**Published:** 2017-11-30

**Authors:** Guoqiang Zhang, Dailin Yu, Bu Sang, Juntao Feng, Lirong Han, Xing Zhang

**Affiliations:** 1 Research & Development Center of Biorational Pesticide, Northwest A & F University, Yangling 712100, Shaanxi, China; gqzhang@shzu.edu.cn (G.Z.); jtfeng@yeah.net (J.F.); zhxing2014@163.com (X.Z.); 2 College of Agriculture, Shihezi University, Shihezi 832000, Xinjiang, China; 3 Agriculture Research institute, Tibet Academy of Agricultural and animal Husbandry Science, Lhasa 850032, Tibet, China; yudailinpz@126.com (D.Y.); sangbu007@foxmail.com (B.S.)

**Keywords:** genome mining, secondary metabolism, *nsdA*, regulator, glycoprotein GP-1

## Abstract

*Streptomyces kanasensis* ZX01 produces some antibiotics and a glycoprotein with antiviral activity. To further evaluate its biosynthetic potential, here we sequenced the 7,026,279 bp draft genome of *S. kanasensis* ZX01 and analyzed all identifiable secondary gene clusters for controlling natural products. More than 60 putative clusters were found in *S. kanasensis* ZX01, the majority of these biosynthetic loci are novel. In addition, the regulators for secondary metabolism in *S. kanasensis* ZX01 were abundant. The global regulator *nsdA* not only controls biosynthesis of some antibiotics, but also enhances production of glycoprotein GP-1 with antiviral activity. This study importantly reveals the powerful interplay between genomic analysis and studies of traditional natural product purification/production increasing.

## 1. Introduction

Actinomyces are an especially abundant source of secondary metabolites, contributing more than half of microbial antibiotics. Moreover, a large number of these metabolites are derived from one genus, *Streptomyces*. Since the publication of the genome of *Streptomyces coelicolor*, researchers were encouraged to mine actinomyces as a source of novel secondary metabolites [1]. Meanwhile, approaches based on genomic or bioinformatics for discovering natural products have been well developed [2]. Genomic information is very helpful to microbial natural products studies, because secondary metabolites of *Streptomyces* are often biosynthesized by complex gene clusters [3]. In addition, it is the genomic information that provides an opportunity for regulating the antibiotic biosynthesis at the molecular level [4]. Studies of metabolic regulation may provide a vital way for increasing production of antibiotics.

*Streptomyces kanasensis* ZX01 was isolated from forest soil around Kanas Lake of China [5]. Its secondary metabolites showed significant and various bioactivities, such as antiviral activity (*Tobacco mosaic virus*, *Cucumber mosaic virus*), antifungal activity (*Cercospora sorghi*, *Exserohilum turcicum*) and antibacterial activity (*Bacillus subtilis*). Glycoprotein GP-1, one metabolite from *S. kanasensis* ZX01, exhibited powerful antiviral activity and wide application prospects [6].

Here we reported all predicted biosynthetic gene clusters for secondary metabolites and abundant regulatory genes from the genome sequence of *S. kanasensis* ZX01. In addition, we evaluated the effect of *nsdA*, a globle regulator, on the production of glycoprotein GP-1 and on the biomass of *S. kanasensis* ZX01.

## 2. Materials and Methods

### 2.1. Strains, Plasmids and Growth Conditions

The wild-type strain of *S. kanasensis* ZX01 (CGMCC 4893) was used in this study. Gause-I agar was used to observe morphology, prepare spore suspensions and plate out conjugations [5]. R2YE medium was used for protoplast transformation [7]. GCBY (glucose 10 g/L, casein hydrolysate 2 g/L, beef extract 1 g/L, Yeast extract 1 g/L, pH 7.2) was used as the liquid medium. All *Streptomyces* cultivations were carried out at 28 °C. *Escherichia coli* ET12567 (A donation from Dr. Keqian Yang), containing a non-transmissible plasmid pUZ8002, was used to propagate unmethylated DNA into *S. kanasensis* ZX01 by conjugation. pDN221 (Invitrogen, Carlsbad, California, USA) was used as the template for the amplification of the kanamycin resistance gene. *E. coli*–*Streptomyces* shuttle plasmid, pKC1139 (A donation from Dr. Keqian Yang), was used to construct the *nsdA* gene-replacement vector [8].

### 2.2. Genome Sequencing, Annotation, and Analysis

The nucleotide sequencing was performed by Chinese National Human Genome Center (CHGC) using a Roche 454 Genome Sequencer FLX (Life Sciences, Branford, CT, USA) and was assembled using Newbler 2.3 (Life Sciences, Branford, CT, USA). Putative protein coding sequences were identified by Glimmer 3.02 [9] and GeneMark [10]. Functional annotation was based on BLASTP results with NR and KEGG databases. The tRNA and rRNA genes were predicted by tRNAscan-SE [11] and RNAmmer [12], respectively. The whole-genome shotgun project was deposited at DDBJ/EMBL/GenBank database under the accession no. LNSV00000000. The draft genome was submitted to antiSMASH for analysis of secondary metabolite gene cluster [13]. 

### 2.3. Quantitative Real-time PCR Analysis

The *S. kanasensis* strain ZX01 was cultured in 100 mL liquid medium for 72 h, then 1 mL γ-butyrolactone (50 mM, CAS No.:96-48-0, Aladdin, Shanghai, China) was added into the liquid medium under aseptic conditions. Control groups were treated with sterile water. One day later, 10 mL of culture were centrifuged briefly to pellet the mycelium, then RNA was purified using an Eastep^®^ Super Total RNA Extraction Kit (Promega, Madison, WI, USA). RNA was treated with amplification grade RNase-free DNaseI (Invitrogen, Carlsbad, CA, USA). In addition, the pure RNA used as the template for cDNA synthesis, then qPCR and data analysis were performed as described by Pullan et al. [14]. The SigA used as the reference gene. The primers of gene fragments were as follow: Ska0065, 5′-GTCGGCCAGGTCCTCGGCTACT-3′ and 5′-TCCGCACCAGGCACTCCACC-3′; Ska1968, 5′-CGCCGCAAGAGCAACAAGAGC-3′ and 5′-CGACAGCCATCTCCAGCAGCAAC-3′; Ska3482, 5′-TGTCC TCGTTCGGCATCAGCG-3′ and 5′-ACAGCACCCACGGCACCACC-3′; Ska3506, 5′-GCCAC TCGGGCCAGGAAGACAT-3′ and 5′-GCAGCACCAGGCTGTTGACGAAC-3′; Ska4292, 5′-ACTTCTGCACGGCCTACCCG-3′ and 5′-AACAGTCCGTTCGACAGCAGGTT-3′; Ska5321, 5′-ACACCGCCTGGGAGCCGTTCTT-3′ and 5′-AGGTCGGCGTGAGGTCGAGGAA-3′; SigA, 5′-CGCCGAGTCCGAGTCTGTGAT-3′ and 5′-CACTGACCATCAGC GTCACAC-3′. 

### 2.4. Construction of nsdA-Null Mutant

Recombinant vector, pKC1139::U5455*kan*^r^D5455 (pRV5455), were generated using overlap PCR technology [15]. The primers of Ska5455 upstream were 5′-CCGGAATTCCGGGCA CCGCAGGTCGAGATGGG-3′ and 5′-GAGATTTTGAGACACGGGCCACGCCGAATCACCG TGTTGCC-3′, downstream primers were 5′-TTACGCTGACTTGACGGGACGCCGACAGCC CGAATCGGTTCCC-3′ and 5′-GCTCTAGAGCGCCGAACCAGCCGAACCACAGC-3′. The kanamycin resistance gene was amplified by PCR using a pairs of primers, 5′-TGGCCCGTGTCTCAAAATCTC-3′ and 5′-CGTCCCGTCAAGTCAGCGTAA-3′. The *nsdA* gene replacement mutant was constructed through a homologous recombination strategy as described by Wang et al. [8].

### 2.5. Purification and Determination of Glycoprotein GP-1

The supernatant liquid from the culture of *S. kanasensis* ZX01 or mutant was filtrated using a 10 kDa ultrafiltration membrane. The filtrate was lyophilized and purified by DEAE-52 column (10 × 2 cm). The 0.1 M NaCl eluent was collected, dialyzed and freeze-dried after water elution. The freeze-dried sample was dissolved in water again and then purified by HiTrap™ ConA 4B column (GE, Branford, CT, USA). The eluent was collected, dialyzed and freeze-dried. Determination of glycoprotein GP-1 was performed by HPLC (Ailgent 1260, Palo Alto, CA, USA) with TSK-GEL G2000SWXL column (7.8 × 300 mm, 5 μm). Flowing phase: ultrapure water; flow rate: 0.5 mL/min; temperature of column: 30 °C; wavelength: 280 nm.

### 2.6. Statistical Analyses

Statistical comparisons were analyzed by one-way analysis of variance (ANOVA). Differences were considered significant when *p* < 0.05.

## 3. Results and Discussion

### 3.1. Sequencing and Gene Annotation of the S. kanasensis ZX01

The size of *S. kanasensis* ZX01 draft genome is 7,026,279 bp, comprising 225 contigs (>500 bp). The genome contains at least 6245 predicted protein coding sequences (CDSs). A total of 4176 (66.87%) CDSs were assigned to known or putative functions, and 2069 (33.13%) CDSs were annotated as hypothetical protein genes. The average of GC content in this genome is 73.88%. The genome also contains seven rRNA operons and 65 tRNA genes. 

### 3.2. Gene Clusters for Secondary Metabolites

The genome of *S*. *kanasensis* ZX01 was mined using bioinformatics tools, namely antiSMASH 4.0, for identifying the gene clusters that are involved in secondary metabolism. Twenty-one biosynthetic gene clusters for secondary metabolic (siderophores, terpenes, phenazine, phosphonate, bacteriocin, ectoine, lassopeptide, thiopeptide, polyketide, nonribosomal peptide, and so on.) were identified by antiSMASH (Table 1). The total length of these gene clusters was almost 535 kb. This analysis told us that 7.61% of the *S. kanasensis* ZX01 genome is occupied by genes related to the biosynthesis of secondary metabolites. Some clusters for the metabolite biosynthesis are conserved in most *Streptomyces* species (e.g., desferrioxamines, isorenieratene, ectoine gene clusters). In these cases, the percent of this genes conserved when compared with known clusters is close to 100%. However, some other gene clusters showed a low conservation (e.g., cluster 11, 12, 14, 15, 16 and 19). Moreover, 40 unclassified putative clusters were identified by antiSMASH (Data not shown). Therefore, the secondary metabolic capability of *S. kanasensis* ZX01 is powerful. Further works would be focused on the function and products of these gene clusters. 

**Figure 1:**
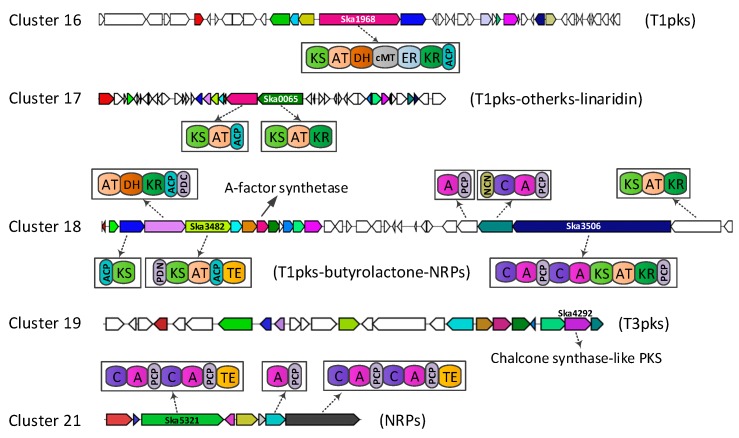
**Proposed PKS/NRPs module and domain organization in five clusters.** PKS: polyketide biosynthase, NRPs: nonribosomal peptide synthetase, KS: ketosynthase, AT: acyltransferase, DH: dehydratase, ER: enoyl reductase, KR: ketoreductase, ACP: acyl carrier protein, cMT: *C*-methyl transferase, PCP: peptidyl carrier protein, C: condensation, A: adenylation, PDN: PKS docking N-term, PDC: PKS docking C-term, NCN: NRPS-COM N-term, TE: thioesterase.

Six putative polyketide synthase (PKS) or nonribosomal peptide synthetase (NRPs) clusters harbored in the genome of *S. kanasensis* ZX01. Two clusters (cluster 16 and 19) contain typical PKS genes (1 type-I and 1 type-III), one cluster (cluster 21) contains NRPs genes, and three complex clusters (cluster 17, 18 and 20) contain PKS or NRPs (Figure 1). We identified a biosynthetic cluster (cluster 18) showing 100% similarity with known micromonolactam (ML) biosynthetic cluster (BGC0000095_c5), which consists of malonyl CoA-acyl carrier protein transacylase (mmlK) and long chain fatty acid CoA ligase (mmlJ). However, the cluster (BGC0000095_c5) depositing in BGC database was a part of whole gene cluster for ML biosynthesis in *Micromonospora* sp. CMS I2-32 [16]. The ML biosynthesis gene cluster was split on 15 different contigs (Accession no.: KC608848-608862). There were some differences between cluster 18 and ML biosynthesis gene cluster on PKS or NRPs domains of other genes. Therefore, a low quality sequencing data will influences the analysis result of gene clusters.

**Figure 2:**
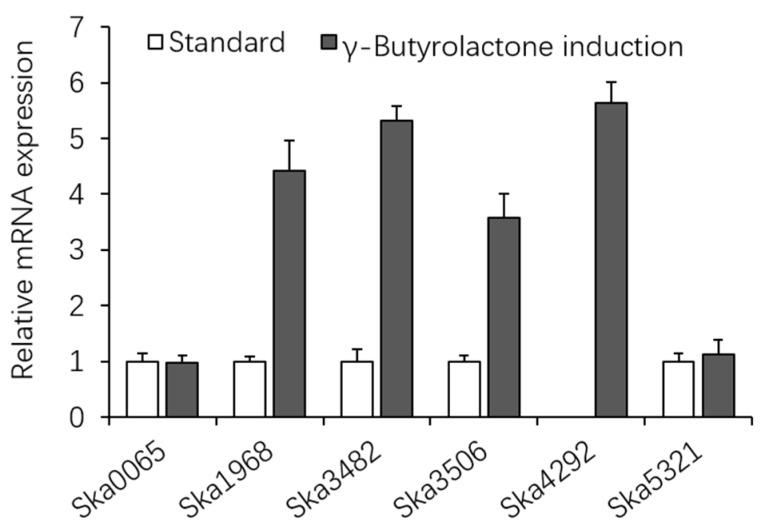
Expression difference of PKS and NRPs genes in *S. kanasensis* ZX01 after γ-butyrolactone induction for 72 h. Ska0065, Ska1968 and Ska3482 are type-I PKS; Ska4292 is type-III PKS; Ska3506 and Ska5321 is NRPs.

The butyrolactone synthetase gene divided cluster 18 into type-I PKS domains and NRPs domains (Figure 1). γ-Butyrolactone (GBL) autoregulators are regarded as microbial hormones that trigger secondary metabolism and morphogenesis in *Streptomyces* [17,18,19]. We found chemical GBL could induce expression of some PKS or NRPs gene. The expression of Ska1968, 3482, 3506 and 4292 increased dramatically under induction condition of chemical GBL (Figure 2). Ska4292 in cluster 17 did not express under general condition (wild strain was inoculated in GCBY liquid medium, then cultivated on shaker at 120 rev/min, 28 °C for 72 h); however, it strongly expressed in the presence of high concentration of chemical GBL. Perhaps, there is a relation between chemical GBL and biological GBL autoinducers. Further experiments are required to confirm this relation.

### 3.3. Regulators for Secondary Metabolism in S. kanasensis ZX01 Genome

Secondary metabolism has a complex regulatory network in *Streptomyces*. Pathway-specific regulatory genes, where each controls one antibiotic biosynthetic pathway, are at the bottom of the regulatory network, such as *actII-orf4*, *redD*, *cdaR* and *mmyR* [20]. Global regulators perform the highest-level regulation and affect the morphological and physiological differentiation. There are at least 466 putative regulators in *S. kanasensis* ZX01 genome, belonging to LuxR, MerR, MarR, AraC, GntR, ArsR family, et al. Some of them, as positive or negative regulators, probably have effects on the secondary metabolism. For examples, *nsdA* negatively regulates the sporulation and antibiotic production in *Streptomyces*. The *nsdA* disruption caused the overproduction of spores and three antibiotics (actinorhodin, calcium-independent antibiotic, epoxycyclopentanone methylenomycin) in *S. coelicolor* [21]. The *Streptomyces bingchengensis* Δ*nsdA* mutant produced more pigments and spores; meanwhile, milbemycin A4 and nanchangmycin were over-produced in the Δ*nsdA* mutant [22].

**Figure 3:**
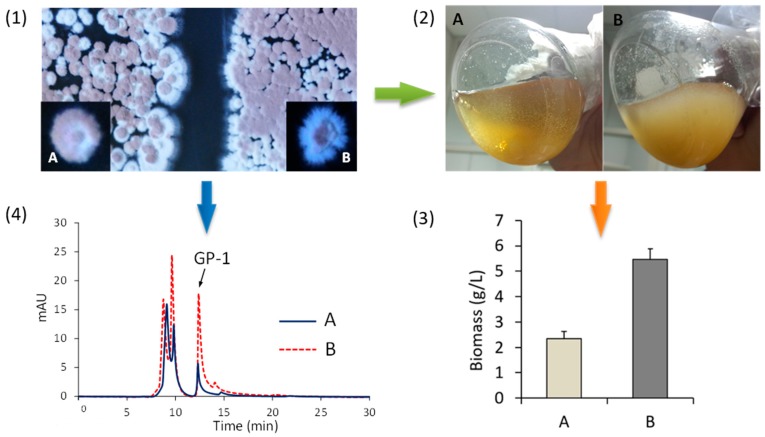
Differences between wild type and mutant of *S. kanasensis* ZX01 in morphology and production of glycoprotein GP-1. (**A**) wild type strain of *S. kanasensis* ZX01; (**B**) *nsdA* disruption mutant in which *nsdA* was replaced by kanamycin resistance gene (*kan*); (**1**) Two strains were cultivated on the Gause-I plate medium for 5 days; (**2**) Two strains was cultivated in GCBY liquid medium for 3 days; (**3**) The differences of biomass between wild type and Δ*nsdA* mutant; (**4**) Determination of glycoprotein GP-1 in wild type and Δ*nsdA* mutant.

In the genome of *S. kanasensis* ZX01 *nsdA* is also present, and affects secondary metabolism and antibiotics. Glycoprotein GP-1 is a secondary metabolite with antiviral activity, producing by *S. kanasensis* ZX01. The Δ*nsdA* mutant of *S. kanasensis* ZX01 produced more spores and aerial hyphae than the wild strain on plate medium or liquid medium, and the production of GP-1 dramatically increased as well (Figure 3). Therefore, the global regulator, *nsdA*, affects colonial morphology and secondary metabolism of antibiotic or non-antibiotic.

At least 46 two-component systems and 7 multi-component systems exist in *S. kanasensis* ZX01 genome. Several two-component systems directly or indirectly affect antibiotics production. The two-component system PhoR-PhoP is the major signal transduction system for phosphate control in *Streptomyces*. In addition, it affects antibiotics production by influencing the transcription of biosynthesis genes, such as, *S. coelicolor* (actinorhodin ACT and prodigiosins RED) [23], *Streptomyces griseus* (candicidin) [24], *Streptomyces rimosus* (oxytetracycline) [25], *Streptomyces natalensis* (pimaricin) [26]. Similar two-component systems, which affect antibiotic production, were predicted in genome of *S. kanasensis* ZX01, including PhoR-P (Ska6032-6034), AfsQ1-Q2 (Ska1698-1697), RapA1-A2 (Ska2296-2297) and CutS-R (Ska4345-4346).

Guanosine tetraphosphate (ppGpp) and pentaphosphate (pppGpp) activate the expression of antibiotic biosynthetic genes, when the supply of amino acids becomes rate limiting for protein synthesis. The gene of (p)ppGpp synthase enzyme *relA* disruption mutant did not produce ACT in *S. coelicolor* [27], but, (p)ppGpp overaccumulation increased ACT and RED production [28]. Ska1310, 3074, 3731 and 3887 probably regulate secondary metabolism of *S. kanasensis* ZX01, because they are all related to (p)ppGpp synthesis. Furthermore, GBL is a signaling molecule as well [29], GBL synthase (ScbA) and receptor (ArpA) affect antibiotic biosynthesis [4]. Ska3485, homologous gene of ScbA, has huge potential for affecting production of secondary metabolites in *S. kanasensis* ZX01.

Many putative regulators for secondary metabolism were found in *S. kanasensis* ZX01 genome. However, most of them are unknown in the function. Hence, mining novel regulatory genes is an important work, especially global regulators, since they serve multiple purposes. Moreover, products of some gene clusters might accurately be labelled ‘stress metabolites’, predicted to combat stresses of a physical (desiccation, low temperature), chemical (low iron) or biological (competition) nature. Therefore, searching for factors that cause the gene clusters to be silent is a focus as well. 

Genome sequencing of *S. kanasensis* ZX01 revealed plenty of novel biosynthetic gene clusters, the majority of them were unexpected and excited. With this available information, there is a need for more genome-guided isolation studies of natural products. Meanwhile, our regulator analysis will facilitate the process of natural product discovery and structure elucidation. Because, studies of regulator may suggest ways of increasing production levels, both at the early stages of characterizing new products and at the level of large-scale industrial production. They may also provide routes to the activation of “silent” gene cluster that are revealed by genome sequencing.

## Figures and Tables

**Table 1:** Putative gene clusters for secondary metabolite in *S. kanasensis* ZX01.

Cluster	Type	GenBank ID	Location		Most Similar Known Biosynthetic Gene Cluster	Similarity (%) *
			From	To		
1	Siderophore	LNSV01000002	180883	189602	Macrotetrolide	33
2	Siderophore	LNSV01000023	1	11484	Desferrioxamine B	100
3	Siderophore	LNSV01000114	10308	18860	-	-
4	Terpene	LNSV01000004	55721	76761	-	-
5	Terpene	LNSV01000004	119146	140210	-	-
6	Terpene	LNSV01000006	39446	61665	-	-
7	Terpene	LNSV01000031	32044	58653	Hopene	61
8	Terpene	LNSV01000035	41851	62261	-	-
9	Terpene	LNSV01000039	39913	59882	Isorenieratene	85
10	Phenazine	LNSV01000013	37297	57785	Marinophenazines	30
11	Phosphonate	LNSV01000030	1	34405	Azinomycin B	6
12	Bacteriocin	LNSV01000030	45699	57147	Enduracidin	6
13	Ectoine	LNSV01000074	2911	13309	Ectoine	100
14	Lassopeptide	LNSV01000003	18630	41325	Collismycin A	7
15	Thiopeptide	LNSV01000094	1	22641	Granaticin	8
16	T1pks	LNSV01000017	13669	61120	Monensin	5
17	T1pks-Otherks	LNSV01000001	32321	84798	-	-
18	T1pks-Butyrolactone-NRPs	LNSV01000041	1	58607	Micromonolactam	100
19	T3pks	LNSV01000059	19531	42521	Merochlorin	4
20	Otherks-Phenazine	LNSV01000010	440	49063	Esmeraldin	64
21	NRPs	LNSV01000098	1	22529	-	-

Secondary metabolite types detected by antiSMASH 4.0: T1pks, Type I polyketide synthase (PKS) cluster; T3pks, Type III PKS cluster; Otherks, cluster containing some PKS modules that does not fit into any other type. NRPs, Nonribosomal peptide synthetase cluster; * The “similarity” means the percentage of the homologous genes in the query cluster that are present in the hit cluster. According to the definition by the antiSMASH, the homologous genes were selected by BLAST e-value < 10^−5^, 30% minimal sequence identity, shortest BLAST alignment covers over >25% of the sequence.
